# Association between Genetic Instability and *Helicobacter pylori* Infection in Gastric Epithelial Dysplasia

**DOI:** 10.1155/2012/360929

**Published:** 2012-12-30

**Authors:** Jin Su Kim, Woo Chul Chung, Kang-Moon Lee, Chang Nyol Paik, Kyeong Soo Lee, Hye Ji Kim, Young Wook Kim, Ji Han Jung, Seung June Noh, Yun Kyung Lee

**Affiliations:** ^1^Department of Internal Medicine, College of Medicine, The Catholic University of Korea, 93 Jungbu-daero, Paldal-gu, Suwon 442-723, Republic of Korea; ^2^Department of Pathology, College of Medicine, The Catholic University of Korea, 93 Jungbu-daero, Paldal-gu, Suwon 442-723, Republic of Korea; ^3^The Research Institute of St. Vincent Hospital, 93 Jungbu-daero, Paldal-gu, Suwon 442-723, Republic of Korea; ^4^Department of Pathology, Samsung Medical Center of Korea, 50 Ilwon-dong, Gangnam-gu, Seoul 135-710, Republic of Korea

## Abstract

*Background*. In gastric carcinogenesis, changes of DNA methylation appear to be an early molecular event, and the genome-wide methylation state is closely correlated with the level of long interspersed nucleotide element-1 (LINE-1) methylation. In this study, we measured LINE-1 methylation level according to genetic instability and evaluated the effect of *Helicobacter pylori* infection on genetic instability in gastric epithelial dysplasia. *Methods*. Total 100 tissue samples of gastric epithelial dysplasia were analyzed. Seven loci that linked to tumor suppressor genes were used to identify significant structural chromosomal aberrations. Microsatellite status was investigated for two different microsatellite marker loci (BAT25 and BAT26). Also, we measured LINE-1 methylation level by combined bisulfite restriction analysis (COBRA-LINE-1) method. *Results*. There were no significant differences of LINE-1 methylation level according to chromosomal/microsatellite instability and *H. pylori* state. In the dysplastic lesions with *H. pylori* infection, LINE-1 methylation level of MSI lesion was significantly lower than that of microsatellite stable (MSS) lesion (40.23 ± 4.47 versus 43.90 ± 4.81%, *P* < 0.01). *Conclusions*. In gastric epithelial dysplasia with *H. pylori* infection, MSI is correlated with reduced LINE-1 methylation level. Coexistence of *H. pylori* infection and MSI might be a driving force of gastric carcinogenesis.

## 1. Introduction

Epidemiological studies in the last decade have established a strong causal relationship between* Helicobacter pylori* (*H. pylori*) infection and gastric cancer, and this bacteria has been classified as a Group I carcinogen by the World Health Organization (WHO) [1–3]. Previously, Correa suggested a human model of gastric carcinogenesis, and he postulated that the development of gastric cancer starts from chronic gastritis to gastric atrophy, intestinal metaplasia, dysplasia, and finally invasive cancer [4]. *H. pylori* infection stimulates cell proliferation in the gastric epithelium and induces apoptosis. It results in imbalance between apoptosis and proliferation and produces alterations or mutations of genes [5, 6]. Eventually, it increases the risk of developing gastric cancer. In the view of this point, the eradication therapy of *H. pylori* would be an attractive therapeutic modality, but it does not prevent the development of gastric cancer in all patients [7, 8]. Researchers are needed to further elucidate how *H. pylori* infection increases the risk of gastric cancer. 

In cancer cell, abnormal DNA methylation is characterized by bidirectional changes—regional CpG island hypermethylation and generalized genomic hypomethylation. Both kinds of changes are observed simultaneously, but these two changes are not reciprocal. They might be independent events [9]. Several studies suggest that genome-wide hypomethylation generally arises earlier, whereas hypermethylation occurs in promoters and is usually a later event. Therefore, global DNA hypomethylation is considered as the hallmark of cancer because the genes vulnerable to aberrant hypermethylation usually are overlapped by the genes targeted by hypomethylation [10–12]. In gastric cancer, global DNA hypomethylation is frequently observed at the very early stage of carcinogenesis [13, 14]. LINE (long interspersed nucleotide element)-1, a highly repeated interspersed human retrotransposon, is ubiquitous and constitutes approximately 17% of the human genome. The level of LINE-1 methylation reflects the genome-wide methylation level, and the reduced level is responsible for the overall losses of DNA methylation. Also, it means regional hypermethylation of specific genes. In gastric carcinogenesis, the levels of LINE-1 methylation decrease from the chronic gastritis to gastric cancer stages, regardless of the status of *H. pylori* infection [15–18].

In this study, we measure LINE-1 methylation level according to chromosomal/microsatellite instability and *H. pylori* status in gastric premalignant lesion. We aim to elucidate mechanisms on how *H. pylori* infection triggers the progression of gastric premalignant lesion to true gastric cancer. 

## 2. Materials and Methods

All tissues were excised by therapeutic endoscopic mucosal resection. The diagnosis of tissue sample was confirmed by two different histopathologists according to the revised Vienna classification; when they disagreed, the tissue sample was excluded from the study. All normal tissues had grossly intact mucosa and were at least 1 cm from the mucosal lesion; they were obtained by gastric biopsy just after an endoscopic mucosal resection. The *H. pylori* status was evaluated according to the histological results (silver stain or CLO test). In the present study, two biopsies were taken both from antrum and corpus after 4 weeks of the endoscopic resection for evaluation of *H. pylori* infection. 

### 2.1. DNA Extraction and Assessment of Loss of Heterozygosity (LOH)

Four-micrometer-thick tissue sections from the dysplasia/cancer and normal tissues were placed on a glass slide and stained with hematoxylin and eosin. The diagnosis of the tissue samples was confirmed by two different histopathologists. Prior to DNA extraction, all the tumor sites were checked for the tumor cell contents ≥70% using a stereomicroscope under a ×40 magnification. To deparaffinize, we utilized a standard series of washes in xylenes and alcohol. Using a 30-gauge needle and a pointed surgical blade, the pathologist performed the microdissection while looking through the microscope. Tissue fragments were deposited into the collection tube. 

LOH was analyzed as described previously [19, 20]. After DNA extraction by standard method, seven loci that linked to tumor suppressor genes were used to identify significant structural chromosomal aberrations. The DNA was amplified by PCR at loci linked to the adenomatous polyposis coli (APC) locus at 5q21 (D5S505), possible tumor suppressor/senescence gene locus at 10p15 (D10S501 and D10S602), the p53 locus at 17p13 (TP53), the BRCA1 locus at 17q21 (D17S855), and the DCC locus at 18q21 (D18S58 and D18S61). 

In brief, 4 mL 30% acrylamide (29 : 1) solution, 2.4 mL 5 × TBE solution, 5.6 mL ddH2O, 200 *μ*L 10% ammonium persulfate, and 10 *μ*L TEMED were blended adequately and poured into the gel then concreted for 1 hour at room temperature. Ten *μ*L of PCR product and 2 *μ*L of loading buffer (95% formamide, 10 mM NaOH, 0.1% bromophenol blue, 0.1% xylene cyanol) were mixed. The mixture was centrifuged for 15 seconds, denatured at 93°C for 3 minutes, bathed in ice for 10 minutes, placed onto a 10% nondenaturation polyacrylamide gel, and separated with 0.5 × TBE buffer for 2 hours at RT and 100 V. 

**Figure 1:**
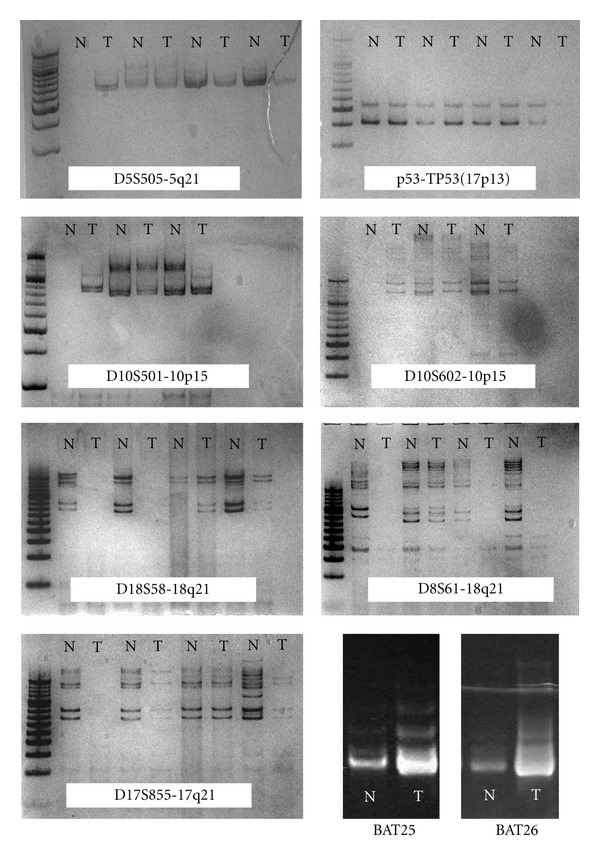
Representative example of loss of heterozygosity (LOH) and microsatellite instability (MSI). Seven loci that linked to tumor suppressor genes were used to identify significant structural chromosomal aberrations (D5S505, D10S501, D10S602, TP53, D17S855, D18S58 and D18S61). Microsatellite status was investigated for two different microsatellite marker loci (BAT25 and BAT26). (NL: normal; T: tumor).

After electrophoresis, the gel was stained using the Bio-Rad Silver Stain kit (Bio-Rad, Philadelphia, PA, USA). Briefly, the gel was fixed with 40% methanol for 30 minutes, oxidized for 5 minutes, rinsed for 5 minutes three times, silver stained for 20 minutes, rinsed for 30 seconds, developed for 1 minute three times, and stopped with 5% acetic acid for 15 minutes. The staining results were analyzed with Gel DocXR (Bio-Rad). Assessment of loss of heterozygosity (LOH) was assigned when a tumor allele showed at least 50% gain or reduction in the relative intensity (Figure 1).

### 2.2. Assessment of Microsatellite Instability (MSI)

DNA samples were amplified using two different oligonucleotide pairs specific for the recommended microsatellite loci BAT25 and BAT26. Primer sequences (Integrated DNA Technologies, Iowa, USA) were: BAT25 (forward 59-TCGCCTCCAAGAATGTAA GT-39 and reverse 59-TCTGCATTTTAACTATGGCTC-39), and BAT26 (forward 59-TGACTACTTTTGACTTCAGCC-39 and reverse 59-ACCATTCAACATTTTTAACCC-39). PCR reactions were performed as described previously. PCR products were run on 8% denaturing polyacrylamide gels at 180 V for 18 hours, and visualized by silver staining (Figure 1).

### 2.3. Assessment of LINE-1 Methylation Status

**Figure 2:**
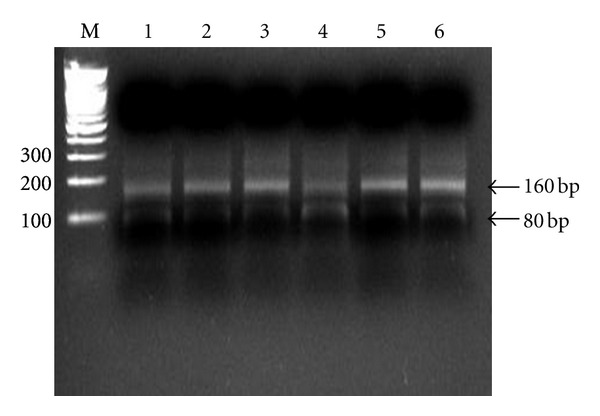
Assessment of LINE-1 hypomethylation status by COBRA LINE-1 method. Calculation was based on the ratio of the digested bands divided by the sum of the digested and undigested bands as described in Materials and Methods section (N: normal; T: tumor).

A modified long interspersed nucleotide elements-combined bisulfite restriction analysis (COBRA LINE-1) method was used to analyze LINE-1 methylation status of the cancers [17, 21, 22]. This method is based on the principle that cytosine in DNA is converted to uracil when DNA is treated with sodium bisulfite, whereas methylated cytosine is protected from the conversion. Thus, the methylated and unmethylated cytosine could be distinguished by digestion with a restriction enzyme that recognizes sequences containing CpG. The extracted DNA was treated with sodium bisulfite and isolated using the EZ DNA methylation kit (Zymo Research, Orange, CA, USA). Bisulfite-treated DNA was amplified by 40 cycles of PCR with two primers, LINE 3 (5V-GYGTAAGGGGTTAGGGAGTTTTT) and LINE 4 (5V-AACRTAAAACCCTCCRAACCAAATATAAA), at an annealing temperature of 50°C. The PCR products were digested with the TaqI restriction enzyme, which recognizes TCGA, for 1 hour at 65°C, and then were separated by electrophoresis on 2% agarose gels. The densities of the digested and undigested bands were obtained by scanning with Gel Doc XR (Bio-Rad, Philadelphia, USA) and scoring with *Quantity One Software* (Bio-Rad, Philadelphia, USA). The ratio of the digested fragments (80 bp) derived from the methylated DNA divided by the sum of the digested fragments and the undigested fragments (160 bp) derived from the unmethylated DNA represents the fractional methylation (expressed as a percentage) at the LINE TaqI site (Figure 2).

### 2.4. Statistical Analysis

For the quantitative variables, the mean and its standard deviation were calculated. For the qualitative variables, the percent and its 95% confidence interval (95% CI) were calculated. We used the *χ* ^2^ test to analyze the association between the *H. pylori* status and other baseline characteristics. For comparison of age and the level of LINE-1 methylation we used the unpaired *t* test. We used the SPSS statistical package (version 12.0.1) for all analyses.

## 3. Results

Total 100 tissue samples (from 61 men, mean age 62.57 ± 6.76 years; 39 women, mean age 63.97 ± 6.34 years) were examined and analyzed. When the gastric epithelial dysplasia (GED) was divided according to the revised Vienna classification, 50 tissues were low-grade (category 3) and 50 were high-grade dysplasia or intramucosal cancer (category 4). Total 54 tissue samples of GED had associated *H. pylori* infection.

### 3.1. LOH in Gastric Epithelial Dysplasia

The incidence of LOH was 83% (83/100) in GED, and the frequencies of LOH were 34% on APC (D5S505), 40% on 10p (D10S501), 48% on 10p (D10S602), 14% on p53 (TP 53), 40% on BRCA1 (D17S855), 51% on DCC (D18S58), and 45% on DCC (D18S61), respectively. According to the classification of chromosomal loss described previously, GED was divided into negative (LOH-negative), low-level (LOH-L; 3 or fewer losses), and high level (LOH-H; 4 or more losses). The incidence of LOH-L was 47% (47/100), whereas LOH-H was 36% (36/100). The frequencies of LOH with *H. pylori* infection were 76.5% (13/17), 48.9% (23/47), and 50.0% (18/36) in LOH-negative, LOH-L, and LOH-H lesion, respectively. There was no significant difference between *H. pylori* infection and LOH state (*P* = 0.06). LINE-1 methylation level of lesions with LOH-negative was not significantly different from that of LOH-positive. Also, LINE-1 methylation level of lesions with LOH-L was not significantly different from that of LOH-H (Table 1). Among the lesions with *H. pylori* infection, LINE-1 methylation level of LOH-L was not different from that of LOH-H (Figure 2).

### 3.2. MSI in Gastric Epithelial Dysplasia

The frequency of microsatellite instability (MSI) was 36% (36/100), and instability rates for the BAT25 and BAT26 were 19% and 29%, respectively. The frequency of BAT25 (+) with *H. pylori* infection was 11%, and BAT26 (+) with* H. pylori* infection was 22%. 

**Figure 3:**
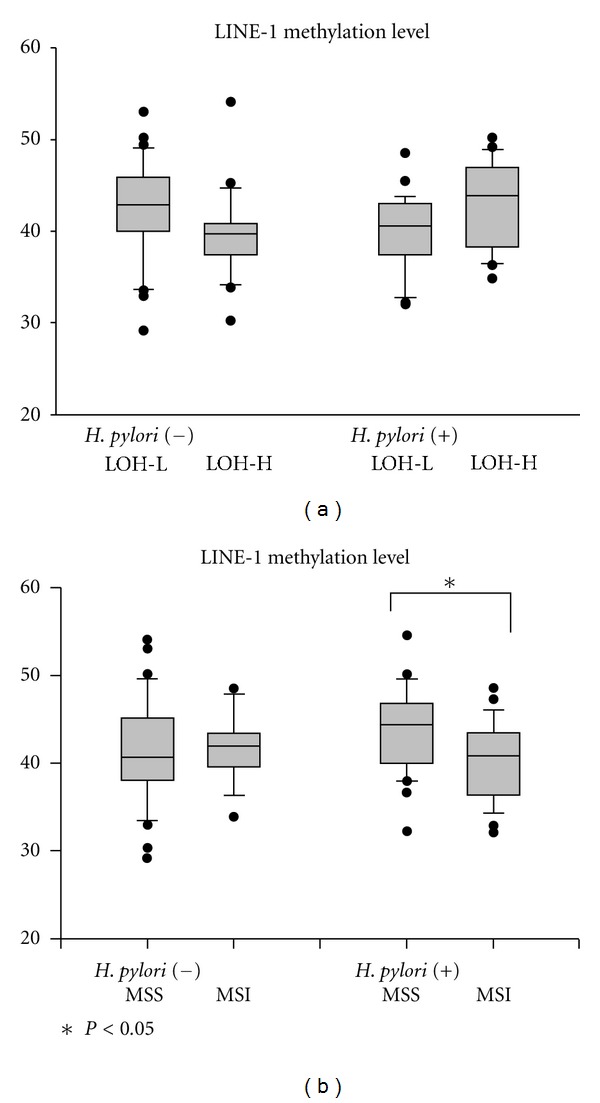
Level of LINE-1 hypomethylation of gastric epithelial dysplasia according to *H. pylori* state and genetic instability. (a) Irrespective of *H. pylori* infection, there were no differences of LINE-1 methylation level between LOH-L and LOH-H. (b) In dysplasia with *H. pylori* infection, LINE-1 methylation level of MSI is significantly reduced than that of MSS. Box plots illustrate median values, 25th and 75th percentiles, and outliers on a linear scale. The unpaired *t* test was applied for nonparametric statistical analysis, and *was considered statistically significant (*P* < 0.05).

LINE-1 methylation level of MSI was not significantly different from that of microsatellite stable (MSS) lesions (Table 1). The frequency of MSI with *H. pylori* infection was 61.1% (22/36), and it was not different from that of MSS lesion (50.0%, 32/64) (*P* = 0.28). In the GED with *H. pylori* infection, LINE-1 methylation level of MSI lesion was significantly lower than that of microsatellite stable (MSS) lesion (40.23 ± 4.47, 43.90 ± 4.81, *P* < 0.01) (Figure 3).

### 3.3. Gastric Epithelial Dysplasia Subgrouped by the Revised Vienna Classification

**Figure 4:**
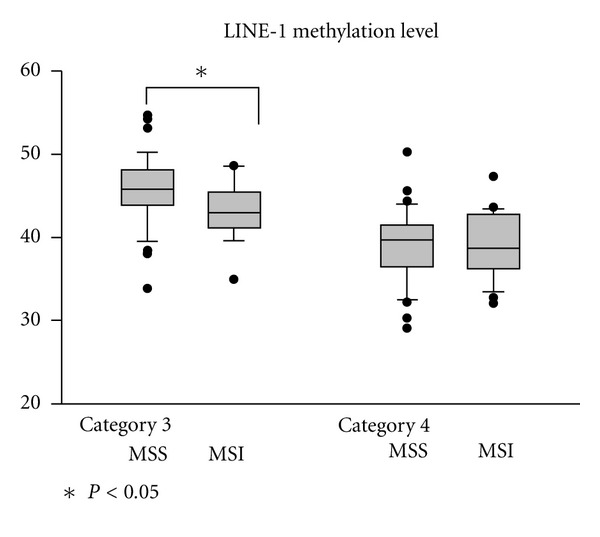
Level of LINE-1 hypomethylation in gastric epithelial neoplasias categorized by the revised Vienna classification. There were no significant differences of LINE-1 methylation level according to the degree of LOH and MSI state. Except in category 3, the lesions with MSI had the lower LINE-1 methylation level than that of MSS. Box plots illustrate median values, 25th and 75th percentiles, and outliers on a linear scale. The unpaired *t* test and one way ANOVA were applied for nonparametric statistical analysis, and *was considered statistically significant (*P* < 0.05).

The tissue samples were divided into two groups according to the revised Vienna classification: low-grade dysplasia (category 3) and high-grade dysplasia/intramucosal cancer (category 4). Twenty six patients with category 3 GED and 28 patients with category 4 GED had associated *H. pylori* infection (*P* = 0.68). The category 4 lesion had lower level of LINE-1 methylation than the category 3 lesion (38.95 ± 4.28 versus 44.93 ± 4.29%, respectively, *P* < 0.01). For categories 3 and 4, the difference in the frequency of LOH-H was not significant (30.0%, 15/50 versus 42.0%, 21/50; *P* = 0.21). The frequencies of MSI positive were 30% and 42.0% (22/51) in categories 3 and 4 (Table 2). In category 3 lesion, LINE-1 methylation level of MSI was significantly lower than that of MSS (43.18 ± 3.66 versus 45.68 ± 4.41%, respectively, *P* = 0.05) (Figure 4).

## 4. Discussion

It is widely accepted that gastric cancer develops through the accumulation of genetic or epigenetic alterations affecting oncogenes and tumor suppressor genes. These alterations involve the mechanisms that control genetic instability. Genetic instability is divided into two categories, chromosomal instability (CIN) and microsatellite instability (MSI) and whether the instability is at the chromosomal or nucleotide level in a lesion [23, 24]. CIN has been recognized as the most common feature of sporadic gastric cancers and CIN phenotype has been reported in up to 84% of gastrointestinal tumors [25], which is compatible with our result. The consequence of CIN is an imbalance in the chromosome number and an increased rate of loss of heterozygosity (LOH). An increased rate of LOH is an important property of CIN, because it accelerates the inactivation of the tumor suppressor genes [26]. In colon cancer model, CIN is an important event in the tumor initiation and progression, and LOH and MSI are inversely correlated [27]. However, in gastric cancer, these are not mutual. In present study, CIN and MSI coincided in 24% of gastric epithelial dysplasia, whereas evidence of both CIN and MSI was lacking in 4%. In the latter cases, it may be associated with the transcriptional silencing of genes by epigenetic alterations.

It is postulated that persistent infection with *H. pylori* initiates chronic inflammation, which induces increased tissue turnover, increased rate of mutagenesis, and genetic instability. However, several researchers suggested that *H. pylori* did not exert direct effects in inducing structural chromosomal aberrations and triggering gastric carcinogenesis [28, 29]. In present study, there were no differences of LINE-1 methylation level, irrespective of *H. pylori *infection and the degree of chromosomal instability (LOH-negative, LOH-L, and LOH-H). It seemed unlikely that *H. pylori* served as a direct inducer of chromosomal instability. However, we cannot rule out the possibility that some cases were negative for *H. pylori* at the time of diagnosis of gastric dysplasia but had suffered *H. pylori* infection previously. The underestimation of the effect of *H. pylori* on chromosomal instability could exist in this study. 

MSI is a molecular phenotype for human cancers with defects in the postreplicative DNA mismatch repair system. *H. pylori* might promote development of gastric carcinoma at least in part through its ability to affect the DNA mismatch repair system and its deficiency resulted in MSI phenotype [30, 31]. It is known that the frequency of MSI in gastric cancer is between 25% and 50% [25]. MSI-positive gastric cancers have been reported to be located in the distal stomach and associated with intestinal-type histology and favorable clinical features [32]. Despite of association with *H. pylori *and MSI, there was no difference of LINE-1 methylation level according to microsatellite state or *H. pylori* infection state in our results. However, in gastric epithelial dysplasia with *H. pylori *infection, MSI had a tendency of the reduced level of LINE-1 methylation. It suggested that *H. pylori *might appear as a cofactor for inducing gastric carcinogenesis. 

To identify MSI, the five suitable markers including BAT25, BAT26, D2S123, D17S250, and D5S346 have been proposed [33]. Nevertheless, we use only two monomorphic mononucleotides (BAT25 and BAT26) in this study. Although the frequency for MSI-positive was considerably similar with the previous study [25], the potential pitfall to define MSI existed because of a huge number and diversity of microsatellite regions in the human genome. However, previous studies show that BAT25 and BAT26 are more sensitive and better markers for microsatellite instability detection than their dinucleotide counterparts [34, 35]. These markers are considered to be sensitive in detecting MSI of tumors and can be testable even in the absence of normal tissue. 

Gastric low-grade dysplasia can progress into an invasive form, but all cases of it do not transform to advanced carcinoma. It has been reported that approximately 15%–30% of low-grade dysplasia progress to high-grade dysplasia or adenocarcinoma [36–38]. To date, there is no doubt that *H. pylori* infection is a major risk factor in the pathogenesis of gastric cancer. The curious problem is which factor determines progression of gastric epithelial lesions. Whether *H. pylori* infection can contribute to the progression of low-grade dysplasia is debatable, and whether the eradication of *H. pylori* infection would reduce the risk of gastric cancer is also controversial. In this point of view, our results are very hopeful. It supports that progression of gastric epithelial dysplasia to true gastric cancer could be blocked after *H. pylori* eradication in the selected cases—MSI positive state. 

In conclusion, MSI-positive gastric epithelial dysplasia with *H. pylori* infection is correlated with reduced LINE-1 methylation level. Coexistence of *H. pylori* infection and MSI might be a driving force of gastric carcinogenesis. To clarify these results, the investigators will conduct a prospective, randomized, and population-based study to determine whether *H pylori* eradication can reduce the incidence of gastric cancer.

## Figures and Tables

**Table 1:** LINE-1 methylation level according to *H. pylori* infection and genetic instability (chromosomal instability and microsatellite instability) in gastric epithelial dysplasia.

		*n*	LINE-1 methylation (%)	*P *value
*Helicobacter pylori *	Positive	54	42.40 ± 4.06	0.34
	Negative	46	41.40 ± 4.21	

Chromosomal instability	LOH (−)	17	40.94 ± 3.97	0.33
	LOH-L	47	42.80 ± 3.39	
	LOH-H	36	41.30 ± 4.13	

Microsatellite state	MSS	64	42.59 ± 4.53	0.09
	MSI	36	40.80 ± 3.44	

LOH: loss of heterozygosity; LOH-L: LOH (+) <3 loci; LOH-H: LOH (+) >4 loci; MSS: microsatellite stable; MSI: microsatellite instable, *statistically significant.

**Table 2:** LINE-1 methylation level according to genetic instability (chromosomal instability and microsatellite instability) in gastric epithelial dysplasias categorized by the revised Vienna classification.

		Category 3 GED			Category 4 GED		
		*n*	LINE-1	*P *value	*n*	LINE-1	*P *value
			methylation			methylation	
Chromosomal instability	LOH (−)	8	44.36 ± 3.43	0.33	9	39.13 ± 4.39	0.58
	LOH-L	27	45.61 ± 2.39		20	38.42 ± 3.47	
	LOH-H	15	44.02 ± 5.79		21	39.35 ± 3.66	

Microsatellite state	MSS	35	45.68 ± 4.41	0.05*	29	38.85 ± 4.56	0.85
	MSI	15	43.18 ± 3.66		21	39.09 ± 3.98	

LOH: loss of heterozygosity; LOH-L: LOH (+) <3 loci; LOH-H: LOH (+) >4 loci; MSS: microsatellite stable; MSI: microsatellite instable, *statistically significant.
